# Suicide prevention training: self-perceived competence among primary healthcare professionals

**DOI:** 10.1080/02813432.2021.1958462

**Published:** 2021-08-03

**Authors:** Pia Solin, Nina Tamminen, Timo Partonen

**Affiliations:** Mental Health Unit, Finnish Institute for Health and Welfare, Helsinki, Finland

**Keywords:** Suicide prevention, training, primary healthcare professionals, self-perceived competence, primary healthcare

## Abstract

**Objective:** The aim is to report the outcomes of the suicide prevention training in terms of the self-perceived impact on the participants.

**Design:** The three-hour training consisted areas of risk and protective factors, screening and evaluating suicide risk, raising concerns and confronting suicidal patients, and treating suicidal ideation in primary healthcare and the associated referral processes.

**Subjects:** The studied participants consisted of general practitioners, nurses, public health nurses and social work professionals.

**Main outcome measures:** Participants assessed their own competence on online form regarding four training areas prior to and two weeks after the training.

**Results:** The response rate was 25%. The self-perceived competence of the healthcare professionals increased in all training areas and in all occupational groups. The healthcare professionals’ competence regarding the *risk and protective factors* training area saw the greatest increase across all professional groups except nurses. There were, however, differences between the groups.

**Conclusion:** Suicide prevention training for primary healthcare professionals did increase the self-perceived competence of the participants in all areas covered by the training. Regular follow-up training is required in order for these improvements to be further developed and retained.

**Key points**
- After the suicide prevention training all participants self-perceived increase in their competence in all training areas.
- The GPs self-perceived most increase in risk and protective factors and nurses in raising concerns and confronting suicidal patients.
- The GPs’ lowest increase was in the area of treating suicidal ideation in primary health care and the referral processes.

## Introduction

Close to 800,000 people take their own life every year. Furthermore, it has been calculated that each death by suicide equates to approximately 20 attempted acts [1]. Young people are among those most at risk, as suicide is the second-leading cause of death for those between the ages of 15 and 29 years globally. While there are high-risk cohorts, however, suicide deaths occur in all age groups, cultures, and population groups [1].

Every suicide has an impact at both the individual and societal level. The long-lasting effects particularly affect those people left behind. It has been estimated that 60 people are directly affected by each suicide death, including family, friends, classmates, and work colleagues. Furthermore, people bereaved by suicide have an elevated risk of suicidal behavior and being affected by mental health conditions such as depression. There are also financial costs for those left behind. The combined burden of costs and losses of earnings incurred through illness-related absences and compassionate leave, as well as the loss of the financial support from the spouse or parent having committed suicide, constitute a significant burden for families [2].

Several studies show that significant numbers of people who kill themselves have visited a general practitioner in the month prior to their suicide [e.g. 3–5]. Furthermore, the study of Piiksi Dahli *et al*. [6] showed that patients with psychological diagnosis had increased number of visits. Still, serious risk factors such as depression and anxiety are often unnoticed together with suicidal ideations in primary health care [7].

Moreover, studies also suggest several explanations as to why the suicidal ideation of these patients remained undetected. For example, patients may be reluctant to discuss their suicidal ideation due to prior negative experiences in this regard. Stigma may also hinder asking for and/or receiving help. Alternatively, the consulting healthcare professional may lack sufficient clinical experience, have insufficient knowledge of mental health conditions, or have underdeveloped communication skills [8,9]. There are studies which suggest that among health care professionals lack of confidence in one’s competence may hinder dealing with psychiatric disorders and suicidal patients [8,10,11].

Furthermore, evidence suggests that some healthcare professionals erroneously believe that voicing concern regarding suicidal intentions increases the likelihood of suicidal acts. For example, in a German survey of primary care practitioners, 23% of respondents stated they would not raise the issue of suicidal ideation among older patients suffering from depression due to the perceived risk of provoking suicidal thoughts among these patients [12]. Further research would suggest, however, that asking about suicidal ideation is not associated with an increase in suicidal acts [13]. To this end, the World Health Organization (WHO) has stressed the importance of acknowledging these issues when planning training for healthcare professionals [1].

The findings of Ahmenadi *et al.* [3] indicate that issues related to mental health and risk of suicide may need more profound assessment, especially in primary healthcare settings. Healthcare providers should, therefore, assess the beliefs, knowledge, and competencies of their staff in these areas and conduct regular training in order to increase awareness of both the warning signs of suicidality and the associated care protocol [14]. Training delivered to primary healthcare professionals, especially to general practitioners, can be an effective tool in suicide prevention. According to the European Psychiatric Association guidelines, training healthcare professionals improves screening for suicidal ideation and suicide risk, and the delivery of adequate care pertaining to depression and anxiety. Such training also changes attitudes and combats stigma [15]. Indeed, Mann et al. [16] found that training for general practitioners focusing on screening for suicide risk decreased patient suicides by 22 to 73%.

A suicide prevention training project for primary healthcare professionals was carried out in Finland in 2017–2018. The project was funded by the Ministry of Social Affairs and Health and developed and executed by experts at the Finnish Institute for Health and Welfare. The suicide prevention training aimed to improve competencies regarding mental health conditions and the associated suicide risks, and to enhance screening and care regarding suicidal intent and mental health disorders.

The aim of this study is to present the before and after results in the self-perceived competence of primary healthcare professionals who participated in suicide prevention training. Also, the social care workers were included in the study as they form an important part of personnel in primary health care. Here, competence refers to their awareness, knowledge, and expertise of the participant, and is assessed in relation to each training area; namely, *risk and protective factors*, *screening and evaluating suicide risk*, *raising concerns and confronting suicidal patients*, and *treating suicidal ideation in primary healthcare and the associated referral processes*.

## Materials and methods

The three hours training included a lecture, discussions of participants’ experiences and videotaped testimonials by an expert of lived experience. A total of 45 training sessions were carried out during the project to a total of 2027 persons of which 37% were primary healthcare professionals and 15% were social work professionals. In this paper, the primary healthcare and the social work professionals are scrutinized. Social work professionals were included in this study as they form an important part of personnel in primary health care.

The content of the training was broken down into four thematic areas: 1) *risk and protective factors*, 2) *screening and evaluating suicide risk*, 3) *raising concerns and confronting suicidal patients*, and 4) *treating suicidal ideation in primary healthcare and the associated referral processes*

The participating primary healthcare professionals were divided into four groups for the purpose of analysis. The three main groups were general practitioners (34%), nurses (40%), and public health nurses (25%). The fourth group consisted of all other primary healthcare occupations (1%), such as physiotherapists and licensed practical nurses. They were not included in the study as their number was low. However, the group of social work professionals (15% of all participants) was included in the study.

The participants were asked to assess their prior competence during the online registration process. Two weeks after the training an online feedback form was sent to all participants (primary health care and social work professionals: *n* = 1048) by e-mail and they were asked to self-assess their competence again. Two remainders were sent. The response rate was 25%.

The participants were asked to answer the following question regarding their self-perceived competence: “What is your own assessment of your competence concerning the following areas of suicide prevention: 1) *risk and protective factors*, 2) *screening and evaluating suicide risk*, 3) *raising concerns and confronting suicidal patients*, and 4) *treating suicidal ideation in primary healthcare and the associated referral processes*?” The respondents scored each area on a Likert-like scale with the answering options as follows: 1= very bad, 2 = bad, 3 = average, 4 = good or 5 = very good. Descriptive statistics were calculated for analysis of the values scored on the Likert-like scales before and after the training as well as for the individual-level changes in the scores.

## Results

**Figure 1.**
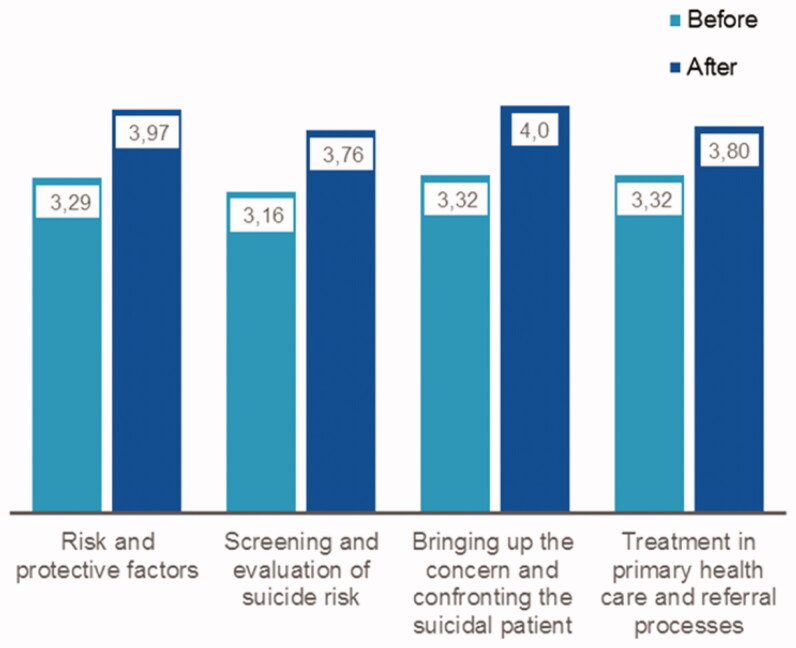
The self-perceived competence before and after the training of all primary healthcare professionals according to training areas on a Likert-like scale of 1 to 5.

Figure 1 presents the change in the self-perceived competence of primary healthcare professionals. The group of primary healthcare professionals includes general practitioners, nurses, public health nurses, and a sub-group of other healthcare professionals, including licensed practical nurses. The average values with their 95% confidence intervals for the self-perceived competence on the areas of suicide prevention before and after the training and their individual-level changes are presented in Table 1.

**Table 1.** The mean (95% confidence interval) values for the self-perceived competence on the areas of suicide prevention before and after the training and their individual-level changes.

	Self-perceived competence concerning the following four areas of suicide prevention											
	Risk and protective factors			Screening and evaluating of suicide risk			Raising concerns and confronting suicidal patients			Treating suicidal ideation in primary health care and the associated referral processes		
Profession	Before	After	Change	Before	After	Change	Before	After	Change	Before	After	Change
General practitioner, *n* = 56	3.20	4.04	0.84	3.20	3.95	0.75	3.21	3.93	0.71	3.18	3.68	0.50
	(3.00–3.39)	(3.87–4.20)	(0.66–1.01)	(3.02–3.38)	(3.83–4.07)	(0.56–0.94)	(2.99–3.44)	(3.80–4.05)	(0.50–0.93)	(2.99–3.37)	(3.44–3.92)	(0.30–0.70)
Nurse, *n* = 76	3.30	3.92	0.62	3.14	3.67	0.53	3.33	4.03	0.70	3.28	3.82	0.54
	(3.14–3.47)	(3.78–4.06)	(0.46–0.78)	(3.01–3.28)	(3.53–3.81)	(0.40–0.65)	(3.15–3.51)	(3.88–4.17)	(0.52–0.87)	(3.08–3.48)	(3.63–4.00)	(0.35–0.73)
Public health nurse, *n* = 63	3.35	4.00	0.65	3.13	3.70	0.57	3.37	4.00	0.63	3.49	3.89	0.40
	(3.18–3.52)	(3.85–4.15)	(0.45–0.85)	(2.94–3.31)	(3.56–3.84)	(0.40–0.74)	(3.17–3.56)	(3.84–4.16)	(0.43–0.84)	(3.31–3.67)	(3.72–4.06)	(0.21–0.59)
Social worker, *n* = 74	3.07	4.04	0.97	2.95	3.62	0.68	3.14	4.00	0.86	3.18	3.78	0.61
	(2.90–3.24)	(3.92–4.16)	(0.82–1.12)	(2.80–3.09)	(3.48–3.76)	(0.53–0.82)	(2.97–3.30)	(3.85–4.15)	(0.70–1.03)	(2.99–3.36)	(3.62–3.94)	(0.45–0.77)

The greatest increases in the self-perceived competence occurred in training area of *risk and protective factors.* This can be seen in all profession groups except nurses. Nurses perceived the greatest increases in their self-perceived competence in training area of *raising concerns and confronting suicidal patients*. Furthermore, the lowest self-perceived increase was assessed in training area of *treating suicidal ideation in primary health care and the associated referral processes* in all other professions except nurses, which estimated lowest self-perceived increases in training area of *screening and evaluating of suicide risk*.

Table 1 also shows that the group of social work professionals, when compared to other profession groups, estimated most increase in their competence in the three quarters of the training areas. These training areas were *risk and protective factors*, *raising concerns and confronting suicidal patients and lastly treating suicidal ideation in primary health care and the associated referral processes*.

The greatest increases in the self-perceived competence among general practitioners occurred in training area *risk and protective factors*. The smallest increase in the self-perceived competence of general practitioners occurred in training area *treating suicidal ideation in primary healthcare and the associated referral processes*. Comparing the results of the group of general practitioners with those of the remaining primary healthcare professionals shows that, when viewed as a separate group, the general practitioners assessed themselves as improving their competence more than the rest of primary healthcare professionals combined. The largest difference was in training area *risk and protective factors*, and the smallest was in training area *treating suicidal ideation in primary healthcare and the associated referral processes*.

**Figure 2.**
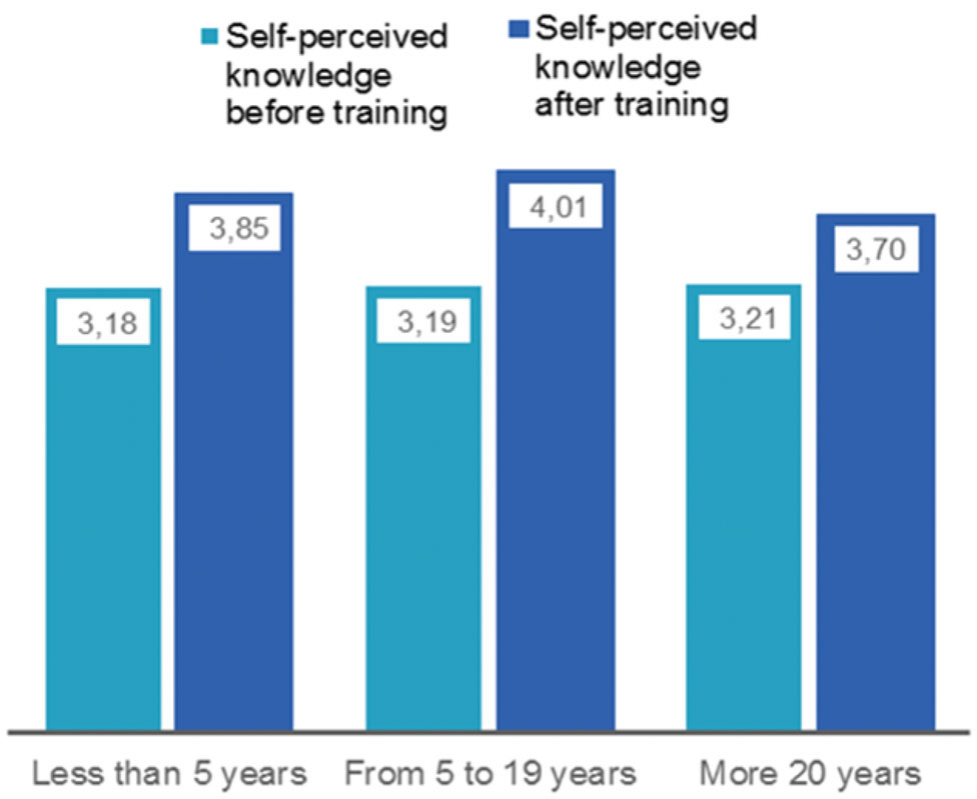
Change of self-perceived competence before and after of the training of general practitioners according to professional experience on a Likert-like scale of 1 to 5.

The training assessment also considered whether the increase in competency of general practitioners was affected by their professional experience, in terms of years of employment in their present role. The employment periods were categorized in three groups: 1) *less than 5 years*, 2) *5–19 years*, and 3) *more than 20 years*. Figure 2 demonstrates that those general practitioners who had been in practice for 5–19 years appeared to benefit most from the training. In contrast, general practitioners with the longest work experience appeared to benefit the least.

## Discussion

The erroneously held beliefs of primary healthcare professionals and their lack of knowledge, experience, and expertise in confronting suicidal patients may be prove to be fatal to suicidal patients. In this training the greatest perceived increase in competence related to training area *risk and protective factors* despite the trainers having considered this area to have been that with which the participants would be most familiar prior to the training. The feedback given by the participants revealed that the discussion of the protective factors was a novel and useful area.

A study by Ritter *et al.* [17] found that general practitioners appear to self-identify as having satisfactory knowledge of suicidality and its management and awareness of at-risk groups. Another study also reported general practitioners’ positive attitudes and relatively high competence in these areas [18]. Nevertheless, the general practitioners in this study by Ritter *et al.* [17] were still reported to have difficulties in assessing suicide risk.

Due to the associated risk of suicidal behavior and increased burden to health care system, training primary healthcare professionals in screening, assessing, and treating mental health disorders and substance abuse is an essential part of suicide prevention [1,6]. Could it be so, then, that even though several studies indicate that primary healthcare professionals still need education and training in the area of suicide prevention and mental disorders, the issue itself is not seen as a top priority in primary healthcare, as Hogan & Goldstein Grumet, [14] suggest. It would appear that despite some progress in recent years in terms of treatment and management strategies for suicide prevention, these strategies are not yet fully utilized [14]. The work by Hogan & Goldstein Grumet [14] also reminds us that a new care model is perhaps needed. For example, there are positive results from the care manager model, where nurses work as care managers following and supporting patients with depression. The care manager provides continuity in care and enables a person-centered care. [19,20] Such a significant change is often challenging, however, especially if it necessitates cultural or clinical change [20].

From all professional groups, social work professionals showed greatest self-perceived increase in three quarters of training areas. In considering the overall increase of primary healthcare professionals’ competence in the aforementioned areas, public health nurses would appear to benefit the most from suicide prevention training, followed by general practitioners and finally nurses.

In the project “Optimizing suicide prevention programs and their implementation in Europe” (OSPI-Europe), the attitudes and confidence of general practitioners when addressing depression and suicide were measured before and after a suicide training program. The results showed that the training improved the attitudes towards suicide prevention and several attitudes towards depression and its treatment. It also increased the general practitioners’ confidence to address depression and suicide in their everyday practice. However, only the general practitioners’ confidence to address depression and suicide was maintained for a longer period. [11] A study by Ramberg *et al.* [21] also found that suicide prevention education is most likely to improve attitudes, clarity, and confidence in the care of suicidal patients. Furthermore, Terpsta *et al.* [22] found that gatekeeper training increases knowledge and confidence in addressing suicidality and consequently recommend suicide prevention training in other sectors than healthcare.

In contrast, some studies show the insufficiency of training in decreasing suicide rates. For example, a study conducted by Morriss *et al.* [23] carried out an educational intervention for primary care staff and mental health workers in contact with suicidal patients. After scrutinizing the suicide rates before and after, the study concluded that interventions intended to improve the assessment and management of suicide may not be sufficient to reduce the population-wide suicide rate. Similar results were seen in a systematic review and meta-analysis conducted by Milner *et al.* [24]. They found that there was no evidence on the efficacy of most of the suicide prevention training interventions studied. Subsequently, they could not recommend initiating any GP suicide prevention initiatives. Even though there are studies [e.g. 23,24] that suggest the training of key personnel in suicide prevention does not affect suicide rates, there are also studies that show training not only increases knowledge, confidence, and skills [e.g. 17] but also reduces suicide rates [25]. As studies [e.g. 10] show that that effects of such training and interventions do not appear to be long-lasting, it is essential the permanence of the competence developed therein is ensured by regular follow-up training sessions [for example 26,27].

## Limitations

There are limitations in the assessment of participants’ self-perceived competence as well as training itself. In order to have an adequate amount of replies from the participants, the online feedback form was kept short. The aim was to collect assessment from overall training together with self-perceived change in competence. Thus assessments relied solely on the self-reporting of the participants and only one question from each training area was asked from the participants.

The data on longitudinal assessment were based on a relatively small sample size. Indeed, even though reminders were sent to the participants twice after the training, the number of assessments remained quite low. Furthermore, long interval effects of training are not assessed.

Furthermore, although some of the participants could have benefited of longer, for example, two-day training, it was estimated that a shorter training would be more accessible to majority.

## Conclusions

This training project indicates that the self-perceived competence of primary healthcare professionals including social work professionals improved in accordance with the suicide prevention training areas. Increase of self-perceived competence may not prevent suicides as such. However, it will most likely increase the self-confidence to trust one’s own observations and raise concern and confront suicidal ideation among patients.

It seems there are very dissenting results regarding the effectiveness of suicide prevention training. Our results and other studies with similar results seem to cautiously suggest that public health centers and private healthcare providers should require their staff to regularly participate in suicide prevention training and thereby maintain their competencies in this area as one measure among others. However, there are several studies showing that suicide prevention training is ineffective. Thus, there are conflicting data whose elucidation might benefit from more research to be conducted. Furthermore, it should also be considered whether suicide prevention training should be combined to other preventive methods [19,20] in order to find out whether more positive and sustained results can be achieved.
